# 肺癌患者自我超越现状及影响因素分析

**DOI:** 10.3779/j.issn.1009-3419.2024.106.16

**Published:** 2024-07-20

**Authors:** Xue YANG, Yu LUO, Lijuan YE, Yingli YU, Daxing ZHU

**Affiliations:** ^1^610041 成都，四川大学华西医院肺癌中心/肺癌研究所; ^1^Lung Cancer Center/Lung Cancer Institute, West China Hospital, Sichuan University, Chengdu 610041, China; ^2^610041 成都，华西护理学院; ^2^West China School of Nursing, Chengdu 610041, China; ^3^610041 成都，四川大学华西医院胸外科; ^3^Department of Thoracic Surgery, West China Hospital, Sichuan University, Chengdu 610041, China

**Keywords:** 肺肿瘤, 自我超越, 希望, 社会支持, 影响因素, Lung neoplasms, Self-transcendence, Hope, Social support, Influencing factor

## Abstract

**背景与目的:**

肺癌患者存在不同程度的自我超越，其能够激发患者的自我意识，推动其积极面对生活中的负性事件，从而改善患者的生活质量及健康结局，但国内关于肺癌患者自我超越的研究鲜有报道，相关影响因素尚未明确。本研究旨在了解肺癌患者自我超越现状并探讨其影响因素，为临床干预决策提供理论依据。

**方法:**

选取2023年9月至2024年2月在四川大学华西医院肺癌中心入院治疗的243例肺癌患者为研究对象；采用一般资料调查表、自我超越量表、Herth希望量表及社会支持评定量表进行调查，并对肺癌患者自我超越的影响因素进行分析。

**结果:**

肺癌患者自我超越总均分为（44.73±8.94）分，希望水平总均分为（37.60±4.98）分，社会支持总均分为（41.31±7.27）分。肺癌患者自我超越与希望水平及社会支持均呈正相关（P<0.001, P<0.001）。学历、希望水平及社会支持是肺癌患者自我超越的影响因素（P<0.05, P<0.001, P<0.05）。

**结论:**

肺癌患者的自我超越处于较低水平，受希望水平与社会支持的影响。医护人员应重视提高肺癌患者的希望水平，开展针对性心理干预，同时引导其增强社会支持感知，从而促进患者自我超越的实现。

## Abstract (en)

**Background and objective:**

Different degrees of self-transcendence exist in lung cancer patients, which can stimulate patients' self-awareness and promote them to face negative events in life positively, thus improving patients' quality of life and treatment outcomes. However, there are few reports on self-transcendence in lung cancer patients in China, and the related influencing factors have not yet been clarified. This study aims to investigate the current situation of self-transcendence in lung cancer patients and explore its risk factors, so as to provide a theoretical basis for clinical intervention decision-making.

**Methods:**

243 lung cancer patients who were admitted to the Department of Lung Cancer Center of West China Hospital, Sichuan University from September 2023 to February 2024 were enrolled as the study subjects; general information questionnaire, self-transcendence scale, Herth hope scale and social support scale were used for the investigation. The influencing factors related to self-transcendence of lung cancer patients were analyzed.

**Results:**

The total mean score of self-transcendence in lung cancer patients was (44.73±8.94); the total mean score of hope level was (37.60±4.98), and the total mean score of social support was (41.31±7.27). Self-transcendence was positively correlated with hope level and social support (P<0.001, P<0.001). Education, hope level and social support were influencing factors of self-transcendence in lung cancer patients (P<0.05, P<0.001, P<0.05).

**Conclusion:**

Self-transcendence in lung cancer patients was at a low level and was influenced by hope level and social support. Healthcare professionals should pay attention to improving the hope level of lung cancer patients, carrying out targeted psychological interventions, and at the same time guiding them to enhance the perception of social support, so as to promote the realization of self-transcendence in patients.

肺癌是世界范围内最常见的恶性肿瘤之一，具有高发病率、高致死率、强转移性等特点，总体5年生存率仅为18%，已成为全球癌症相关死亡的主要原因^[1⇓-3]^。由于非特异性症状和不适当的早期筛查方法，肺癌的确诊通常比大多数的癌症更晚，患者确诊时多合并其他疾病，加之术后严重创伤以及周期性放化疗所引起的副作用，导致患者疾病负担严重，心理压力巨大，继而容易出现情绪失调与行为紊乱，严重影响患者生活质量和预后^[4,5]^。自我超越通常源于健康和疾病经历，是在面对不良生活事件或疾病时，个体通过内省的方式重建自身对外界事物的看法，调整人生目标，从而获得心理健康和幸福感知^[6]^。研究^[7,8]^显示，高水平的自我超越能够极大程度地激发患者的自我意识，推动其积极面对生活中的负性事件，促进精神健康，从而改善患者的生活质量及健康结局。目前国内外研究^[9,10]^均表明癌症患者存在不同程度的自我超越，且受自尊、自我控制感及自我效能等因素的影响，但国内关于肺癌患者自我超越的研究鲜有报道，相关影响因素尚未明确，难以为临床医护人员开展针对性干预提供指导。因此，本研究旨在了解肺癌患者自我超越现状进行调查并分析其影响因素，以期为医护人员制定有效策略以实现肺癌患者的自我超越提供理论依据。

## 1 资料与方法

### 1.1 研究对象

选取2023年9月至2024年2月四川大学华西医院收治的243例肺癌患者为研究对象。纳入标准：（1）年龄≥18岁；（2）经病理确诊为原发性肺癌；（3）意识清楚，认知功能正常；（4）自愿参与本研究。排除标准：（1）病情危重或存在严重功能障碍（视力听力严重受损），无法配合完成调查者；（2）既往存在严重精神疾病史。采用Kendall^[11]^样本量估算方法，样本量为自变量数量的5-10倍。本研究共选取自变量11个，考虑20%的样本缺失率，计算得出样本量为66-132例，最终实际共纳入243例肺癌患者。本研究已通过四川大学华西医院伦理委员会的伦理审查，审查号为2023年审（985号）。

### 1.2 研究工具

#### 1.2.1 一般资料调查表

调查表由研究者自行设计，包括性别、年龄、婚姻状况、学历、职业状态、患病年限、医疗费用支付类型、家庭人均月收入等人口学特征。

#### 1.2.2 自我超越量表（self-transcendence scale, STS）

源量表由Reed^[12]^编制，我国学者张晶等^[13]^进行文化调适并修订后形成中文版本，目前已广泛应用于患者自我超越水平的评估。STS采用Likert 4级评分，逐级计为1分（一点也不）-4分（非常多），共15个条目，得分越高，表明自我超越水平越高，总得分以45分为临界值，≤45分为低水平，>45分为高水平。本研究中STS的Cronbach’s α系数为0.948。

#### 1.2.3 Herth希望量表（Herth hope index, HHI）

源量表由美国学者Herth^[14]^研制，用于简要评估患者的希望水平，经我国学者赵海平等^[15]^汉化修订后形成12个条目的中文版本。HHI各条目与采取积极的行动、对现实和未来的积极态度、与他人保持亲密关系等3个维度相对应。采用Likert 4级评分，逐级计为1分（非常不同意）-4分（非常同意），其中条目3与条目6为反向赋分，其余条目均为正向赋分，得分越高，表明希望水平越高，根据总得分可将希望水平归为3类：低水平（12-23分）、中等水平（24-35分）、高水平（36-48分）。本研究中HHI的Cronbach’s α系数为0.904。

#### 1.2.4 社会支持评定量表（social support rate scale, SSRS）

源量表由国内学者肖水源^[16]^编制，共10个条目，分别对应3个维度：主观支持、客观支持和对社会支持的利用度。采用Likert 4级评分法，总得分的范围为12-66分。SSRS在本研究中的Cronbach’s α系数为0.779。

### 1.3 资料收集方法

征得科室管理人员同意后，由两名经过培训的护理调查员严格根据纳入排除标准筛选研究对象后进行调查。采用问卷星形式进行资料收集，开展调查时由调查员采用统一指导语面对面向调查对象详细陈述调查的目的、意义及注意事项，征得其知情同意后方可实施。问卷由调查对象独立完成填写，如调查对象对问卷内容存在疑惑，由调查员及时解答和澄清，对于因故无法独立填写者，由调查员复述问卷条目内容并代为填写。为保证问卷的准确性和完整性，问卷回收后调查员及时核查有无错漏，必要时请患者修改或补充资料。

### 1.4 统计学方法

采用SPSS 26.0软件进行数据分析。在统计描述部分，计数资料采用频数（百分比）表示，计量资料采用均数±标准差表示。采用独立样本t检验或方差分析比较肺癌患者自我超越得分在一般资料中的差异；应用Pearson相关性分析及多元线性回归分析探索自我超越与希望水平及社会支持等变量之间的关系。P<0.05为差异有统计学意义。

## 2 结果

### 2.1 一般资料

本研究共调查及纳入243例肺癌患者，其中男性167例（68.7%），女性76例（31.3%）；年龄：<60岁127例（52.3%），≥60岁116例（47.7%）；未婚6例（2.5%），已婚224例（92.2%），离异/丧偶13例（5.3%）；学历：小学62例（25.5%），初中82例（33.7%），高中/中专50例（20.6%），大专24例（9.9%），本科及以上25例（10.3%）；职业状态：在职41例（16.9%），无业110例（45.2%），离退休92例（37.9%）；患病年限：<1年179例（73.7%），1-5年54例（22.2%），>5年10例（4.1%）；医疗费用支付类型：自费4例（1.6%），新农合74例（30.5%），城镇医保52例（21.4%），职工医保98例（40.3%），商业保险15例（6.2%）；家庭人均月收入：<3000元54例（22.2%），3000-5000元63例（25.9%），>5000元126例（51.9%）。

### 2.2 肺癌患者自我超越、希望水平及社会支持得分情况

本研究中肺癌患者自我超越、希望水平及社会支持总均分分别为（44.73±8.94）、（37.60±4.98）和（41.31±7.27）分。各维度详细得分见表1。

**表1:** 肺癌患者自我超越、希望水平及社会支持得分（n=243, Mean±SD）

Index	Total average score	Item average score
Self-transcendence	44.73±8.94	2.98±0.60
Hope index	37.60±4.98	3.13±0.41
Temporality and future	12.79±1.68	3.20±0.42
Positive readiness and expectancy	12.12±1.89	3.03±0.47
lnterconnectedness	12.68±1.80	3.17±0.45
Social support	41.31±7.27	4.13±0.73
Subjective support	24.55±4.57	6.14±1.14
Objective support	9.56±2.66	3.19±0.89
Utilization of support	7.21±1.99	2.40±0.66

### 2.3 肺癌患者自我超越的单因素分析

将243例肺癌患者的一般资料纳入自我超越得分的组间比较。单因素分析结果显示，肺癌患者自我超越在学历及职业状态方面具有统计学差异（P<0.05），其他变量均无统计学差异（P>0.05）（表2）。

**表2:** 肺癌患者自我超越的单因素分析（n=243, Mean±SD）

Item	n	Score	Statistical value	P
Gender			1.448^a^	0.149
Male	167	45.29±9.27		
Female	76	43.50±8.09		
Age (yr)			-1.346^a^	0.180
<60	127	43.99±9.69		
≥60	116	45.55±8.00		
Marital status			1.542^b^	0.146
Unmarried	6	40.17±11.32		
Married	224	45.01±8.78		
Divorce/widow	13	41.92±10.22		
Educational background			4.096^b^	0.003
Primary school	62	43.42±8.04		
Junior high school	82	42.70±9.53		
High school/vocational school	50	47.40±7.19		
Junior college	24	49.00±8.75		
Undergraduate or above	25	45.20±10.19		
Occupational status			3.342^b^	0.037
Employed	41	45.22±9.48		
Unemployed	110	43.17±8.84		
Retired	92	46.37±8.58		
Years of illness (yr)			1.109^b^	0.331
<1	179	44.43±9.17		
1-5	54	44.98±7.84		
>5	10	48.70±10.21		
Medical expense payment type			1.820^b^	0.126
Self funded	4	43.07±8.02		
New rural cooperative medical care	74	44.12±9.07		
Urban medical insurance	52	45.90±9.45		
Employee medical insurance	98	41.75±5.19		
Commercial insurance	15	48.20±8.98		
Per capita monthly income of households (¥)			2.530^b^	0.082
<3000	54	43.91±8.15		
3000-5000	63	43.03±8.10		
>5000	126	45.93±9.53		

^a^: t-value; ^b^: F-value.

### 2.4 肺癌患者自我超越、希望水平与社会支持的相关性分析

肺癌患者自我超越总分与希望水平总分及社会支持总分均呈正相关（r=0.654, P<0.001; r=0.406, P<0.001）。

### 2.5 肺癌患者自我超越影响因素的多元线性回归分析

多元线性回归分析结果显示，学历、希望水平与社会支持是肺癌患者自我超越的影响因素（P<0.05, P<0.001, P<0.05）（表3）。

**表3:** 肺癌患者自我超越影响因素的多元线性回归分析（n=243）

Variables	B	SE	β	t	P
Constant	-3.865	3.471	-	-1.114	0.267
Educational background	0.766	0.344	0.108	2.228	0.027
Hope index	1.059	0.094	0.590	11.220	<0.001
Social support	0.166	0.066	0.135	2.536	0.012

Independent variable assignment: Education level (primary school=1, junior high school=2, high school/vocational school=3, junior college=4, undergraduate and above=5); Occupational status (employed=1, unemployed=2, retired=3); both the level of hope and social support scores will be input based on the original values.

## 3 讨论

### 3.1 肺癌患者自我超越处于较低水平

243例肺癌患者自我超越得分为（44.73±8.94）分，总体处于较低水平，与贺丽莎^[17]^的研究结果相近。分析原因可能是：一方面，受传统文化熏陶，国内患者在应对生活负性事件时通常较为内敛隐忍，多采用自我消化的方式化解消极情绪，难以主动表达内心真实感受，心理压力随着疾病进程而显著增加，患者自我超越意识得不到有效唤醒^[18]^；另一方面，由于肺癌的隐匿性及快速进展性，多数患者在确诊时已进展至中晚期，复杂的疾病治疗方案、严重的症状负担及沉重的经济负担与患者渴望回归正常生活的期望相冲突，患者无法很好地接纳自身，从而极易产生焦虑、抑郁等负性情绪，导致自我超越的实现受阻^[19]^。因此，临床医护人员应关注和重视肺癌患者的自我超越水平，积极开展针对性心理干预，引导患者挖掘自身潜在力量，以积极心态应对负性事件，促进自我超越的实现。

### 3.2 肺癌患者自我超越的影响因素

#### 3.2.1 学历

本研究结果显示，学历是肺癌患者自我超越的影响因素（P<0.05），即学历越高，该人群的自我超越水平越高，与康婵娟等^[20]^的研究结果相似。分析其原因，文化程度较高的肺癌患者，其获取及理解肺癌相关健康保健或自我护理知识的能力较强，能够更加积极地面对疾病所带来的负担^[21]^；此外，在看待问题的态度和行为方面，文化程度高的肺癌患者更易进行深度思考，乐于接纳自身，心理状态较为稳定，内在资源不易受外界影响，能够通过自我心理调节努力超越现状^[22]^。因此，医务工作者应重点关注文化程度较低的肺癌患者，实施个性化的健康教育，从而提升患者认知理解水平，促进自我超越。

#### 3.2.2 希望水平

希望是一种多维动态的生命力，是指个体期望通过充分调动内在资源以实现美好未来及人生目标的信念^[23]^。本研究结果显示，希望水平是肺癌患者自我超越水平的保护因素（P<0.001），与Haugan^[24]^的研究结果一致。高希望水平的患者对自己实现目标的能力充满自信，能够充分调动内在资源和力量，在逆境中保持充沛精力，在肺癌的诊断和治疗过程中，能积极主动地参与治疗决策，从而解决实际问题，实现自我超越^[25]^。此外，希望水平较高的患者，其内在资源更为稳定，在面临手术或放化疗所引起的不良刺激时，更倾向于以积极的心态进行自我调整，自我感受负担相应下降^[26]^。因此，在临床护理过程中，医护人员应重视对肺癌患者希望水平的评估，开展个性化健康教育与心理疏导活动，调动患者治疗积极性，增强其希望感知，从而促进希望水平与自我超越的良性互动。

#### 3.2.3 社会支持

社会支持是在负面事件和压力背景下，由家庭、朋友或其他社区网络提供的精神或物质援助，其形式包括情感支持、工具支持、信息支持、陪伴支持及尊重支持^[27,28]^。本研究结果显示，社会支持是肺癌患者自我超越的保护因素（P<0.05），说明社会支持水平越高，患者越容易感知自我超越，这与Matthews等^[29]^的研究结果一致。坚实的社会支持作为一种积极因素，不仅能够使患者提升自我价值感，推动其乐观地面对自身病情，避免消极情绪的沉积，还能凭借组织或公会进行资金筹集、开展社工活动等举措缓解其经济及心理压力，保障其生活质量^[30]^。肺癌治疗手段繁杂，患者生存期跨度较大，通常对于情感、物质支持具有较高的需求。基于此，医护人员应明确肺癌患者的需求类型并提供相应的社会支持干预，同时鼓励患者主动寻求多元化支持，扩展支持来源，促进自我超越。

综上所述，肺癌患者的自我超越处于较低水平，且学历、希望水平及社会支持是肺癌患者自我超越的独立影响因素。在临床实践中医护人员应关注和重视肺癌患者自我超越水平，针对患者不同特征及实际需求制定个性化干预方案，帮助患者树立个人信念，提升希望水平，改善社会支持状况，从而促进自我超越的实现。本研究仅收集了单中心数据，样本代表性受限，今后可考虑扩大样本量，深入探讨肺癌患者自我超越的动态变化轨迹；未来研究也可纳入更多人口学及心理学指标，进一步探索自我超越的预测因素，为制定促进自我超越的干预策略提供参考。
